# Spontaneous dissection of the superior mesenteric artery and the right hepatic artery: a case report

**DOI:** 10.1186/1752-1947-4-87

**Published:** 2010-03-16

**Authors:** Nicolas C Buchs, Pierre Charbonnet, Frank Schwenter, Christoph D Becker, Philippe Morel, Sylvain Terraz

**Affiliations:** 1 Clinic for Visceral and Transplantation Surgery, Department of Surgery, University Hospitals of Geneva, Geneva, Switzerland; 2 Department of Radiology, University Hospitals of Geneva, Geneva, Switzerland

## Abstract

**Introduction:**

Isolated spontaneous dissection of the superior mesenteric artery is a very rare condition. Endovascular stent placement has been proposed recently for selected cases, which has led to some good clinical results.

**Case presentation:**

We report a case of spontaneous dissection of the superior mesenteric artery spreading to the origin of a right hepatic artery in a 48-year-old Chinese man. He benefited from the placement of an endovascular stent that yielded excellent results.

**Conclusion:**

Endovascular stent placement is a good alternative treatment for dissection of the superior mesenteric artery. We propose an algorithm for the management of this rare condition.

## Introduction

Isolated spontaneous dissection of the superior mesenteric artery (SMA), without the involvement of the abdominal aorta, is a very rare condition [1,2]. Fewer than 80 cases have been reported in the literature since the first case described by Bauerfeld in 1947 [3].

The majority of patients who present with hypovolemic shock or peritonitis are treated surgically [4,5], while asymptomatic patients have occasionally been managed conservatively [1,6,7]. Recently, endovascular stent placement has been proposed for selected cases, and this has led to some good results [2,8-11].

We report a case of spontaneous dissection of the SMA that had spread to the origin of the right hepatic artery. We describe a relatively new and promising therapeutic approach in this case report. Finally, we propose an algorithm for the management of an isolated dissection of the SMA.

## Case presentation

A 48-year-old Chinese man was admitted to the Emergency Department at the University Hospitals of Geneva with a sudden onset of severe epigastric pain and an episode of bilious vomiting that subsided completely within a few hours. He had no relevant medical history except hypercholesterolemia. He also denied any history of arterial hypertension, diabetes mellitus or any recent trauma. On physical examination, our patient was pale and sweaty with a temperature of 36.8°C, a regular heart rate of 84 beats per minute and a blood pressure of 130/70 mmHg. An examination of his abdomen revealed epigastric tenderness without signs of peritonism. His white blood cell count was 8.3 × 10^9 ^cells/L and his C-reactive protein was 3 mg/L. His liver function tests, serum lipase and amylase levels were normal. His cardiac troponins were 0.012 μg/L and his electrocardiogram result was normal.

**Figure 1:**
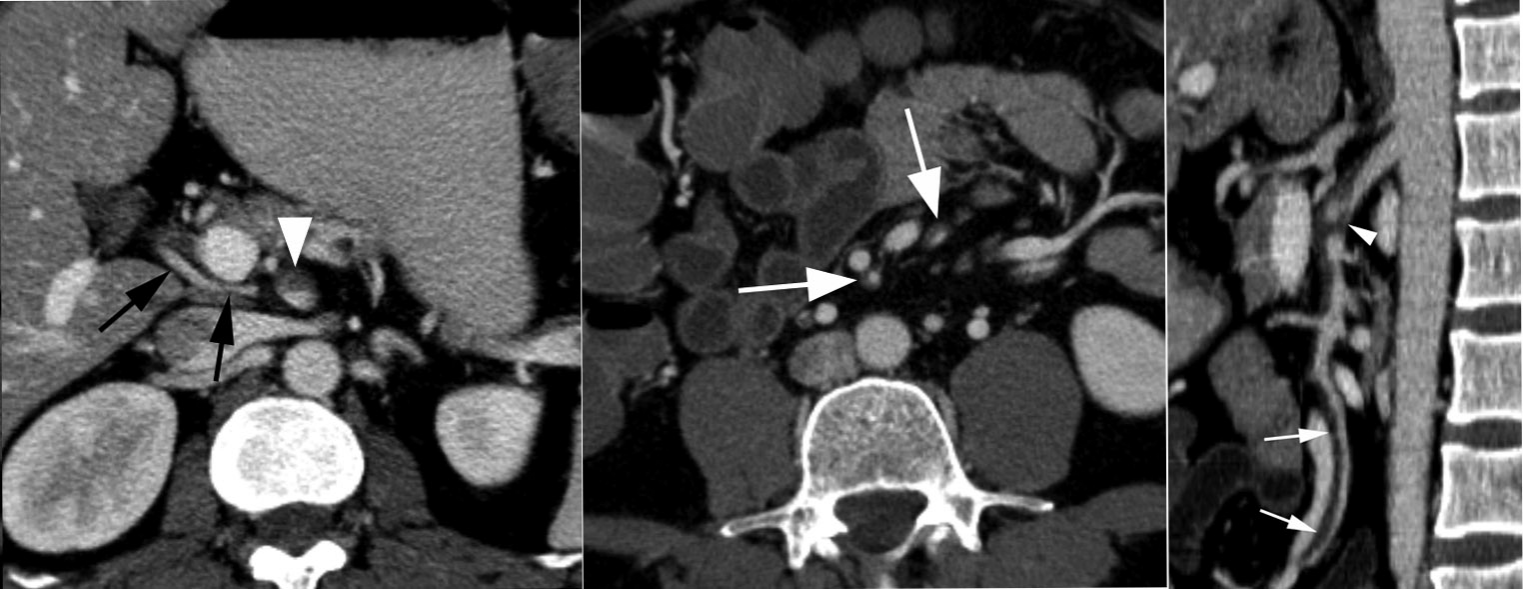
**Contrast-enhanced computed tomography during the portal phase**. (A) The axial image at the level of the proximal superior mesenteric artery shows a dissection with a mural thrombus (white arrowhead), which is associated with minimal inflammation of the mesentery. Note the accessory right hepatic artery (black arrows) that runs behind the portal vein. (B) The axial image at a lower level shows extension of the dissection to distal arterial branches (white arrows). The small bowel and the colon have a normal appearance. (C) The curved multi-planar reconstruction along the main trunk of the superior mesenteric artery shows the origin of the dissection approximately 1 cm from its ostium and distal extension to a jejunal artery (white arrows). The true lumen of the superior mesenteric artery is severely compressed by a dilated, partially thrombosed false lumen (white arrowhead).

**Figure 2:**
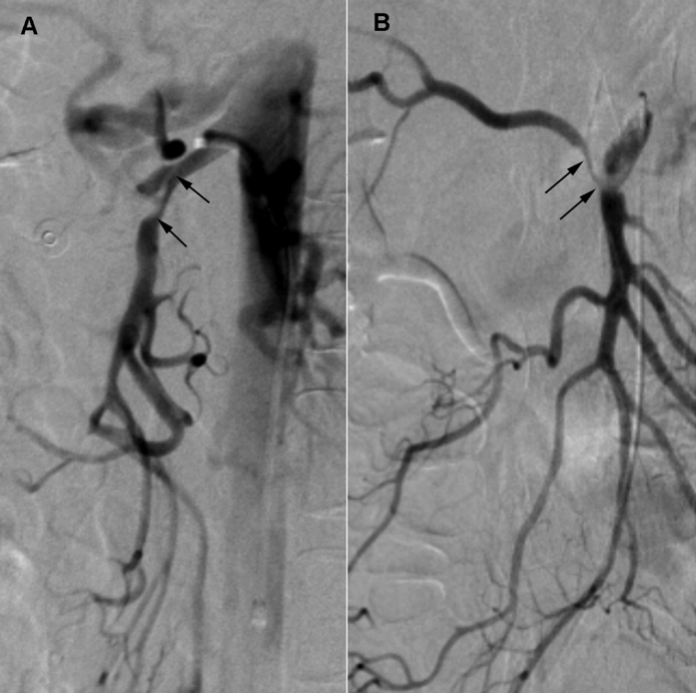
**Digital subtraction angiography of the superior mesenteric artery with a 5F Cobra catheter**. (A) The lateral arteriogram shows the entry site of the false lumen and confirms the compression of the true lumen by an intimal flap (black arrows). (B) The posteroanterior arteriogram demonstrates the extension of the dissection to the origin of the accessory right hepatic artery (black arrows), which is also markedly narrowed. Note the patency of the distal arterial branches.

A contrast-enhanced computed tomography (CT) scan revealed an enlarged and irregular diameter of the SMA with a mural thrombus and without signs of bowel ischemia or ascites. A curved multi-planar reconstruction of the SMA showed a dissection of the proximal SMA with extension into the jejunal and ileal arteries (Figure 1). Selective angiography of the SMA was performed using a 5F transfemoral Cobra catheter (Cordis, Roden, The Netherlands) which confirmed the diagnosis of dissection with compression of the proximal SMA (Figure 2). Interestingly, an accessory right hepatic artery arose from its false lumen, while distal arterial branches of the SMA were patent.

**Figure 3:**
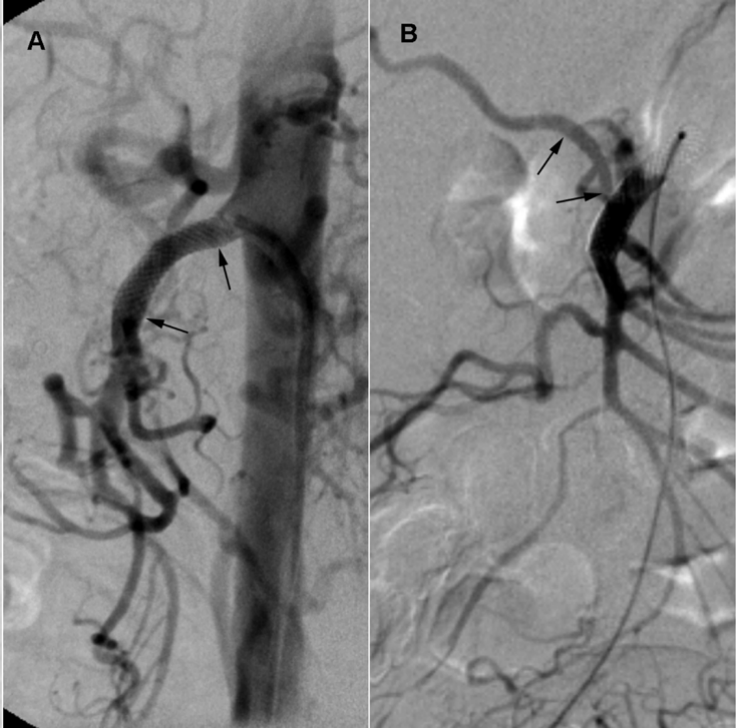
**Digital subtraction angiography of the superior mesenteric artery after the placement of a metallic endoprosthesis**. (A) The lateral arteriogram shows the final position of the endoprosthesis at the proximal part of the superior mesenteric artery (black arrows) with a complete re-canalization of its true lumen. (B) The posteroanterior arteriogram further demonstrates the reopening of the accessory right hepatic artery (black arrows).

Since our patient was asymptomatic and did not show any abdominal complications, we considered percutaneous stent placement instead of surgery in order to treat the dissection and to prevent its progression. Oral informed consent was obtained from our patient before the procedure. Using the same transfemoral route, an 8F 55 cm RDC guiding catheter (Cordis, Roden, The Netherlands) was introduced into his abdominal aorta. A 0.035-inch guide wire (Terumo, Leuven, Belgium) was eased into the true lumen of his SMA through the stenosis after an intra-arterial infusion of 5000 IU of heparin. After measuring the diameter of the SMA using a selective arteriogram, a self-expandable metallic endoprosthesis (Wallstent; Boston Scientific, Galway, Ireland), 10 mm in diameter and 20 mm in length was placed over the entrance of the false lumen and the obstructing intimal flap (Figure 3). A control angiogram showed a patent true lumen with good flow in all the branches of the SMA, including the accessory right hepatic artery.

After the procedure, our patient commenced treatment with long-term 100 mg aspirin, which was combined with an oral loading dose of 300 mg clopidogrel, followed by 75 mg clopidogrel daily for 28 days. A Doppler ultrasonography was performed at 24 hours then seven days after the stent placement. This showed patency of the endoprosthesis with normal spectral Doppler waveforms in the main distal branches of the SMA. Our patient was discharged eight days after the procedure and has remained completely asymptomatic during the following three months.

## Discussion

The SMA is the second most frequent site of isolated spontaneous peripheral arterial dissection after the carotid artery. This condition was first described in 1947 by Brauenfeld [3].

Its management can be categorized into three different eras. Before the 1970s, all patients died, while from the 1970s until the early 2000s surgery remained the only successful option. In 1998, Yasuhara *et al. *[12] reported the first cases of patients with spontaneous SMA dissection who recovered fully without undergoing surgery. In 2000, Sparks *et al. *initiated a conservative approach with anticoagulation [13]; while Leung *et al. *described percutaneous endovascular treatment [9].

This evolution in the management of SMA is concordant with the improvement in CT resolution and interventional radiology which have enabled the diagnosis of dissection to be made more frequently, thus encouraging the application of non-invasive procedures. Sakamoto *et al. *[6] reported detailed morphological models of dissection, while distinguishing between four types of lesions. On this basis they established a set of treatment modalities for isolated spontaneous SMA dissection.

**Figure 4:**
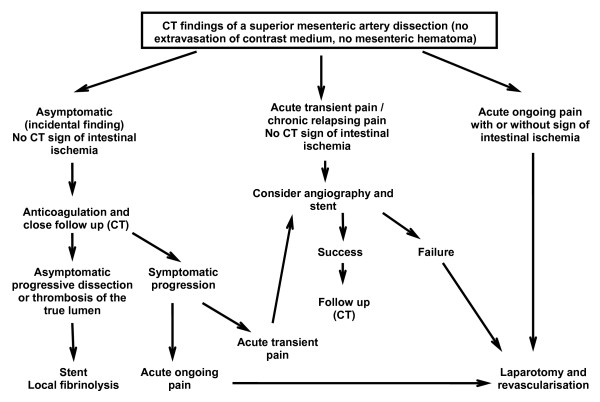
**Algorithm proposed for the management of superior mesenteric artery dissection**.

In our case, we were confronted with a healthy patient. We had to decide how to manage a limited SMA dissection extending to the origin of his right hepatic artery. His pain ceased spontaneously within a few hours and no signs of contrast extravasation were observed. No associated vascular anomalies, such as aneurysm and stenosis, or intestinal ischemia were documented. Finally, we developed an algorithm (Figure 4) for a therapeutic approach based on symptomatic presentation and CT imaging scan.

We differentiate between three modalities of clinical presentation: an asymptomatic patient whose dissection is an incidental finding, a patient with acute transient pain or chronic relapsing pain (non-continuous pain), and a patient with acute ongoing pain (continuous pain).

In the first type of presentation, we suggest using anticoagulation and close follow-up with serial CTs. We deem this approach valid as CT angiography has been proven to be as accurate as catheter angiography in evaluating the location and extent of the dissection [14,15]. As recommended for carotid artery dissection [16], anticoagulation should prevent thrombosis of the true lumen and embolic events. There is no current recommendation for the intervals between the scans [1]. Very close follow-up must be continued as asymptomatic progressive dissection or thrombosis of the true lumen may occur.

For symptomatic patients (transient or ongoing pain), treatment is mandatory [17] even if pain may only be related to inflammation around the dissecting SMA and does not necessarily correspond to acute intestinal ischemia [14]. However, it has been well-demonstrated recently that symptomatic patients have had complete resolution of their abdominal pain after a stent placement [17].

Our patient belongs to the category of patients who develop acute transient pain, which ceases spontaneously, and who have no signs of ischemia on CT. With this clinical presentation, we believed that angiography should be performed, and endovascular treatment of the lesion should be considered. According to Ozaki *et al. *[18], conservative management or minimally invasive procedures may be alternatives to surgery in such cases. Close follow-up is also recommended with particular attention to any new abdominal pain. We had some concern regarding our patient's right hepatic vascularization. Fortunately, angiography documented collaterals and preserved perfusion through his right hepatic artery after the stent placement. The post-operative period was uneventful and no derangement of his liver function tests was observed. It is obvious that this form of management can only be considered if a high level of competency in radiological interventions is locally available.

## Conclusion

Surgery is mandatory for patients with continuous pain and/or signs of intestinal ischemia on CT. For patients with transient pain, however, a percutaneous approach performed in specialized centers should be considered. Finally, a conservative approach using anticoagulation and close follow-up is recommended for incidental findings.

## Consent

Written informed consent was obtained from our patient for publication of this case report and any accompanying images. A copy of the written consent is available for review by the Editor-in-Chief of this journal.

## Competing interests

The authors declare that they have no competing interests.

## Authors' contributions

NCB, PC and FS analyzed and interpreted the data and wrote the manuscript. ST performed the stent placement and the arteriography. PM and CDB were major contributors in writing and correcting the manuscript. NCB and PC have contributed equally to this work. All authors read and approved the final manuscript.
